# Machine learning in predicting cardiac surgery-associated acute kidney injury: A systemic review and meta-analysis

**DOI:** 10.3389/fcvm.2022.951881

**Published:** 2022-09-15

**Authors:** Zhe Song, Zhenyu Yang, Ming Hou, Xuedong Shi

**Affiliations:** ^1^Qinghai University Medical School, Xining, China; ^2^Qinghai University Affiliated Hospital Intensive Care Unit, Xining, China

**Keywords:** thoracic surgery, machine learning, cardiac surgery-associated, acute kidney injury, meta-analysis

## Abstract

**Background:**

Cardiac surgery-associated acute kidney injury (CSA-AKI) is a common complication following cardiac surgery. Early prediction of CSA-AKI is of great significance for improving patients' prognoses. The aim of this study is to systematically evaluate the predictive performance of machine learning models for CSA-AKI.

**Methods:**

Cochrane Library, PubMed, EMBASE, and Web of Science were searched from inception to 18 March 2022. Risk of bias assessment was performed using PROBAST. Rsoftware (version 4.1.1) was used to calculate the accuracy and C-index of CSA-AKI prediction. The importance of CSA-AKI prediction was defined according to the frequency of related factors in the models.

**Results:**

There were 38 eligible studies included, with a total of 255,943 patients and 60 machine learning models. The models mainly included Logistic Regression (*n* = 34), Neural Net (*n* = 6), Support Vector Machine (*n* = 4), Random Forest (*n* = 6), Extreme Gradient Boosting (*n* = 3), Decision Tree (*n* = 3), Gradient Boosted Machine (*n* = 1), COX regression (*n* = 1), κNeural Net (*n* = 1), and Naïve Bayes (*n* = 1), of which 51 models with intact recording in the training set and 17 in the validating set. Variables with the highest predicting frequency included Logistic Regression, Neural Net, Support Vector Machine, and Random Forest. The C-index and accuracy wer 0.76 (0.740, 0.780) and 0.72 (0.70, 0.73), respectively, in the training set, and 0.79 (0.75, 0.83) and 0.73 (0.71, 0.74), respectively, in the test set.

**Conclusion:**

The machine learning-based model is effective for the early prediction of CSA-AKI. More machine learning methods based on noninvasive or minimally invasive predictive indicators are needed to improve the predictive performance and make accurate predictions of CSA-AKI. Logistic regression remains currently the most commonly applied model in CSA-AKI prediction, although it is not the one with the best performance. There are other models that would be more effective, such as NNET and XGBoost.

**Systematic review registration:**

https://www.crd.york.ac.uk/; review registration ID: CRD42022345259.

## Introduction

Cardiac surgery-associated acute kidney injury (CSA-AKI) is a common complication following cardiac surgery, with its morbidity rising due to the increasing demand of cardiac surgery worldwide. The prevalence of cardiac surgery ranges from 0.5 to 500 per million in developing countries. Cardiac and vascular procedures are common risk factors in CSA-AKI, with 3% of the patients requiring renal replacement therapy. According to the Kidney Disease Improving Global Outcomes (KDIGO) criteria (1), AKI is defined as a sudden deterioration of renal function within a period of hours to days, and is characterized by the decrease of serum creatinine (SCr) levels, estimated glomerular filtration rate (eGFR), blood urea nitrogen (BUN), and urine output, with high morbidity and mortality. It can be divided into three stages based on either a decrease of urine output or an increase of SCr (2). AKI often requires high treatment costs, and inappropriate management for it can lead to chronic kidney disease (CKD) or end-stage renal disease (ESRD) (3). AKI induces not only short-term adverse events but long-term poor outcomes such as fluid and electrolyte disturbance. Even mild AKI is associated with poor patient survival according to the KDIGO (1) analysis. An analysis of recovery patterns after AKI shows that 41.2% of the patients could not have their renal function recovered before hospital discharge.

Cardiac and vascular surgery is one of the common risk factors for AKI. The incidence of CSA-AKI reaches up to 40% (4). Blood dynamics alteration following cardiac surgery causes a decrease in renal blood perfusion, and subsequently reduces eGFR, leading to necrosis of glomerular epithelial cells, which underlies the pathogenesis of CSA-AKI (5, 6).

Machine learning refers to computer simulation or implementation of human behavior to endow the computer with the ability of self-improvement so as to be capable of complex multitasking. It covers multiple disciplines such as mathematics, statistics, and computer science, and has been widely used in scientific research and industry. In recent years, machine learning has also been widely applied in disease prediction, and multiple studies on the use of machine learning in CSA-AKI prediction have been reported. However, its predictive value lacks evidence-based support. Therefore, we conducted this systematic review and meta-analysis to evaluate the predictive value of machine learning for CSA-AKI so as to provide evidence-based support for its clinical application (7).

## Methods

This meta-analysis is carried out in strict accordance with The Preferred Reporting Items for Systematic Reviews and Meta-Analyses (PRISMA) 2020 statement, which has been preregistered on PROSPERO (Registration No. CRD420222345259).

### Literature search

Cochrane Library, PubMed, EMBASE, and Web of Science were searched from inception to 18 March 2022. Search items were designed based on the combination of medical subject headings and free words, without language and region restriction. A literature search was conducted by Zhe Song (detailed search strategy is shown in Supplementary File 2).

### Inclusion and exclusion criteria

Studies meeting the following criteria were included:

1. Randomized controlled trial (RCT), prospective cohort study, nested case-control study, case-control study, and registration data on patients with cardiovascular diseases who had undergone heart surgery such as heart valve replacement and cardiac contrast.
2. A complete predictive model was established;
3. Published in English.

Exclusion criteria were:

1. Study unrelated to CSA-AKI or only reported risk factors;
2. Containing no outcome measures related to the effectiveness of a predictive model (e.g., RFC, sensitivity, specificity, accuracy, confusion matrix, etc.);
3. Other study design: case reports, letters, conference summaries, reviews, etc.;
4. Incomplete data or data unavailable.
5. AKI staged using KDIGO (1) serum creatinine criteria; cardiac function graded *via* the American Heart Association guideline and ESC 2021 guideline (8, 9).

### Literature search

All articles identified were imported into EndNote X9. Titles and abstracts of the articles were browsed following duplicate removal, and the full-texts of the remaining articles were retrieved and read to identify eligible studies. Literature search and screening were processed by two reviewers (SZ and YZY) independently, any disagreements were settled by a third reviewer (HM). The articles searching a flow chart are presented in Supplementary materials. Pieces of literature, which contain unclear information or missing critical data, were excluded from our study.

### Data extraction

The data extraction form was designed according to the Modified CHARMS checklist (10), which mainly included: name of the first author, publication date, nation, duration of data hiring, study design (prospective and retrospective), types of validation (external, internal, random split, and time split), and sample size (total number, developments, and testing cluster). The development set was defined as all data sets other than the test set in this study due to the unclear description in each study.

### Risk of bias assessment

We used the prediction model risk of bias assessment tool (PROBAST) (11) and the external prognostic validity model to assess the risk of bias in the included studies. PROBAST is a risk of the bias assessment tool designed for systematic reviews of diagnostic or prognostic prediction models. It contains four domains: participants, predictors, outcome, and statistical analysis. Items under each domain can be filled as “yes,” “probably yes,” “probably no,” “no,” and “no information,” depending on the characteristics of the study. If a domain contains at least one item filled as “no” or “probably no,” it would be graded as high risk. A domain with all the items filled as “yes” or “probably yes” would be graded as low risk. The overall risk of bias would be graded as low risk when all the domains are graded as low risk. The risk of bias assessment was performed by two reviewers independently.

### Statistical analysis

We calculated and reported descriptive statistics to summarize the characteristics of the models. For prediction models that were examined in more than two independent datasets (excluding the model development dataset), a random-effect meta-analysis was performed to estimate the performance and accuracy. Prediction models, which were internally validated through bootstrapping or cross-validation and were externally validated in only two independent datasets, were also considered. We followed a recently published framework of meta-analysis for prediction models. If a measure of uncertainty (standard error or 95% confidence interval) was not available for the mean C-index, a formula was used to approximate the standard error of the mean C-index based on the number of events and number of participants. All data analyses were performed using the R software (Version 4.1.1).

## Results

### Study selection

There were 1,909 articles identified [Cochrane (*n* = 133), PubMed (*n* = 33), Embase (*n* = 231), Web of Science (*n* = 1,512)]. After removing 220 duplicates, titles and abstracts of the remaining 1,689 articles were browsed, and 38 studies (12–49) were finally included. A PRISMA flow diagram of the study selection process is shown in Supplementary File 4.

### Characteristics of included studies

A total of 139,444 participants were involved, with 116,499 in the validation set. Data were collected from 12 countries. Among the included studies, 25 (about 66%) have been published in recent 5 years (2017–2022), indicating that research in the field of the machine learning-based prediction model has been a hotspot in recent years, and is of great value and significance.

These were 60 prognostic models for CSA-AKI included, 12 external validation models, and 7 random sampling validation models. The types of these 60 prognostic models include: Logistic Regression (12–16, 19–22, 24–35, 37–49) (*n* = 34), Neural Net (15, 17–19) (*n* = 6), Support Vector Machine (15, 16) (*n* = 4), Random Forest (15, 16, 30, 40) (*n* = 6), Extreme Gradient Boosting (15, 16, 49) (*n* = 3), Decision Tree (15, 16) (*n* = 3), Gradient Boosted Machine (19) (*n* = 1), COX regression (19) (*n* = 1), κ Neural Net (19) (*n* = 1), and Naïve Bayes (19) (*n* = 1). Characteristics of included studies are shown in Supplementary File 1.

### Quality assessment

**Figure 1:**
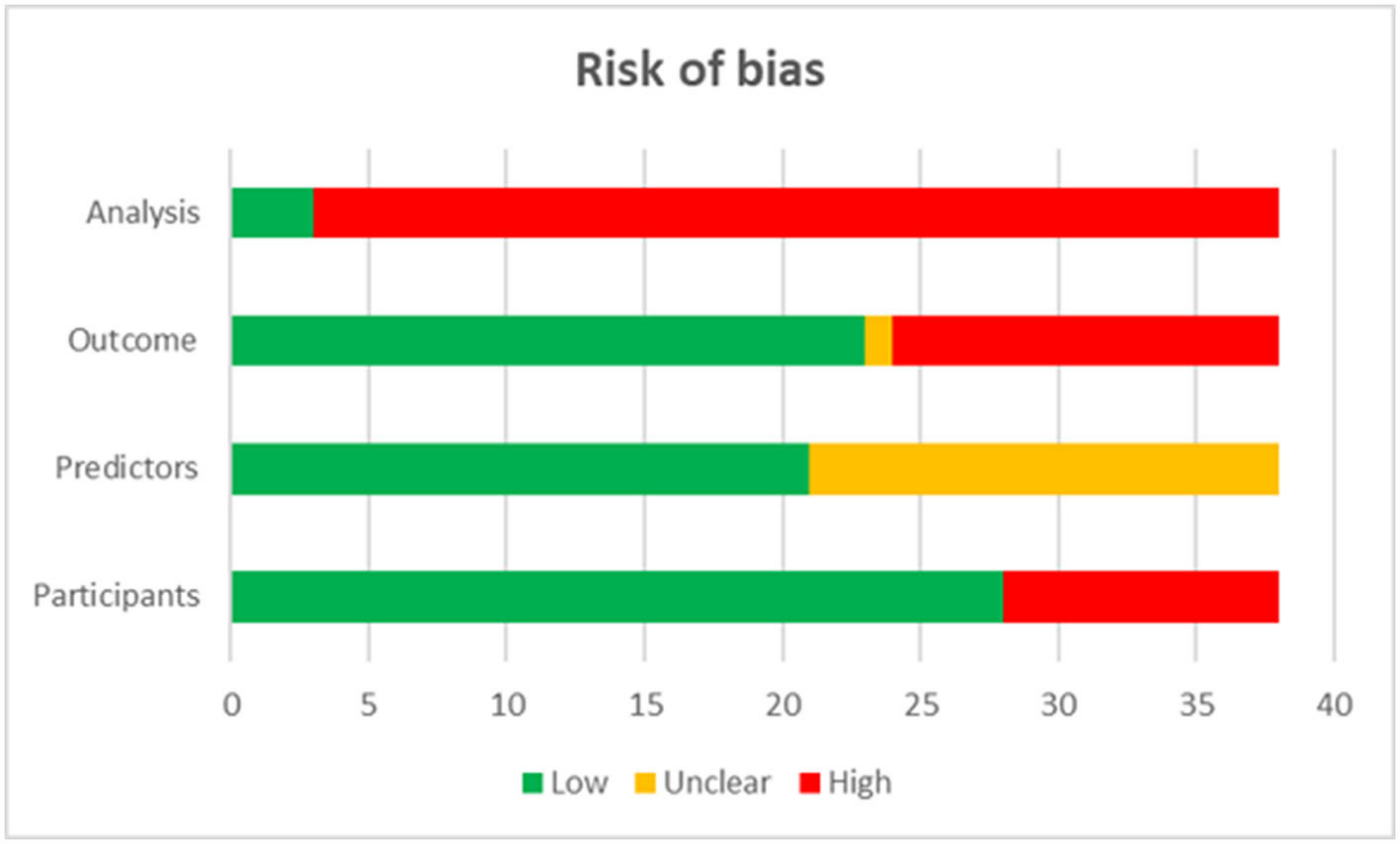
Quality assessment of included studies.

The quality assessment showed that 92.11% of included studies were graded as high risk in the domain of analysis, 36.84% were graded as high risk in the domain of outcomes, and 26.32% in that of participants (Figure 1).

### Predictors

**Figure 2:**
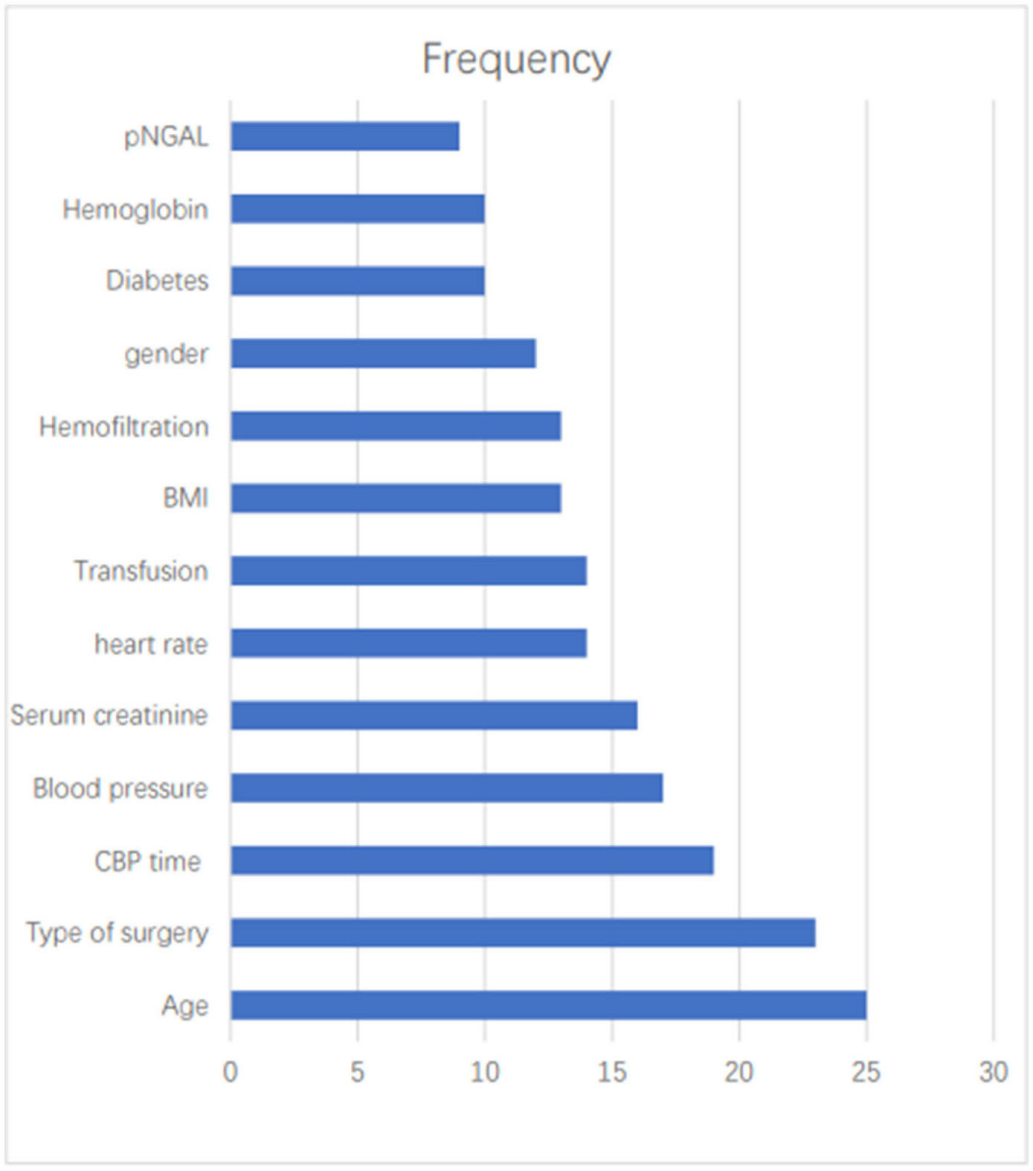
A frequency bar chart of predictors.

The most commonly used predictors were Age (*n* = 25, 41.67%), Types of surgery (*n* = 23, 38.33%), CBP time (*n* = 19, 31.67%), Blood pressure (*n* = 17, 28.33%), SCr (*n* = 16, 26.67%), heart rate (*n* = 14, 23.33%), Transfusion (*n* = 14, 23.33%), BMI (*n* = 13, 21.67%), Hemofiltration (*n* = 13, 21.67%), gender (*n* = 12, 20.00%), diabetes (*n* = 10, 16.67%), Hemoglobin (*n* = 10, 16.67%), pNGAL (*n* = 9, 15.00%) (Figure 2).

### Training set and test set accuracy

**Figure 3:**
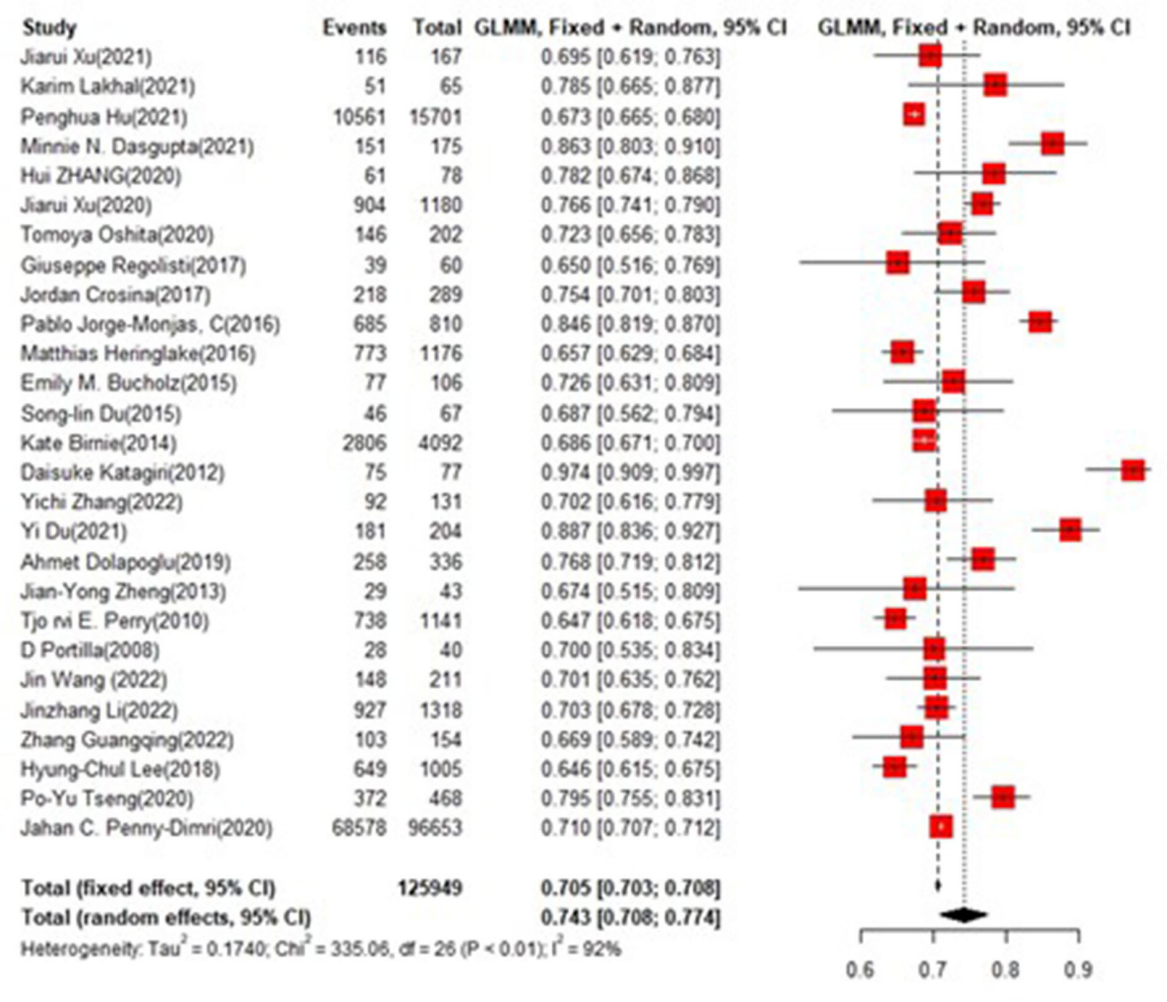
Accuracy of the machine learning-based model in the training set: logistic regression.

**Figure 4:**
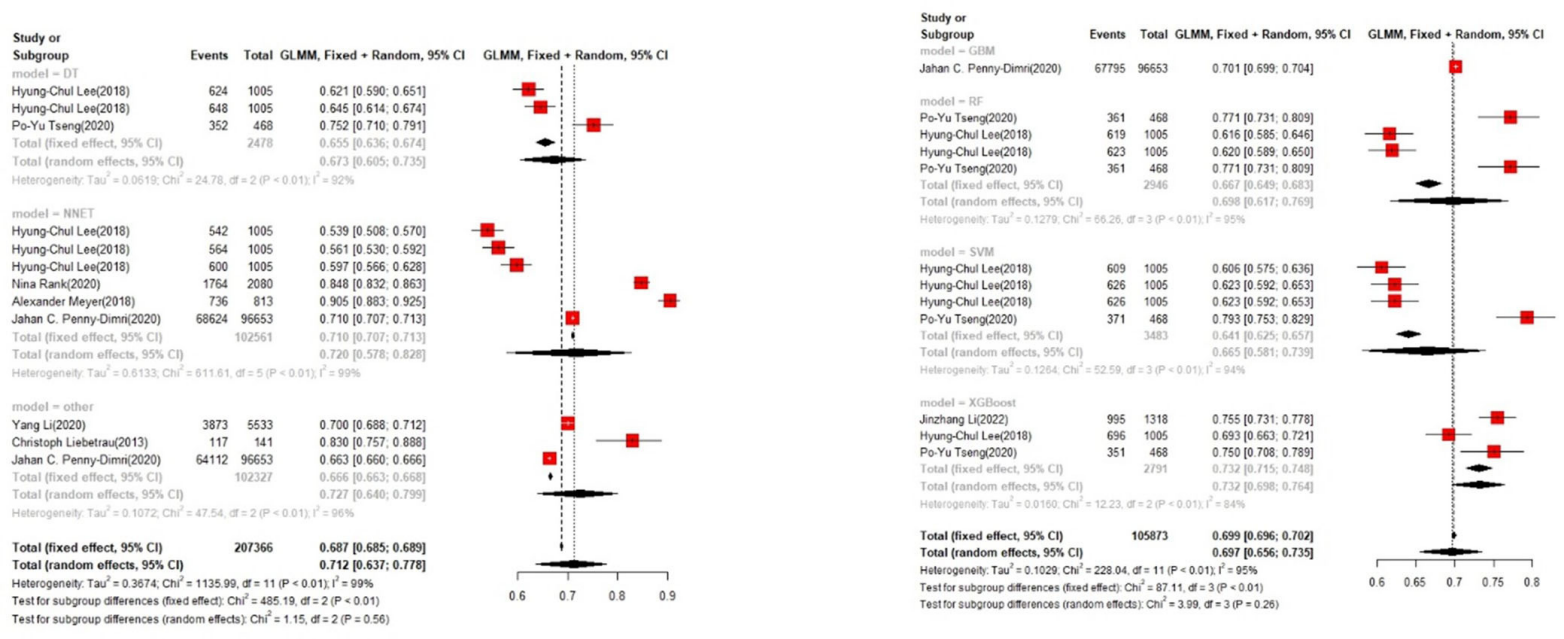
Accuracy of the machine learning-based model in the training set: other models.

In the training set, the logistic regression model was the most commonly applied [*n* = 27, accuracy = 0.705 (0.703, 0.708)]. XGBoost showed to be of the best performance [*n* = 3, accuracy = 0.732 (0.715, 0.748)], with large modeling sample size (Figures 3, 4).

**Figure 5:**
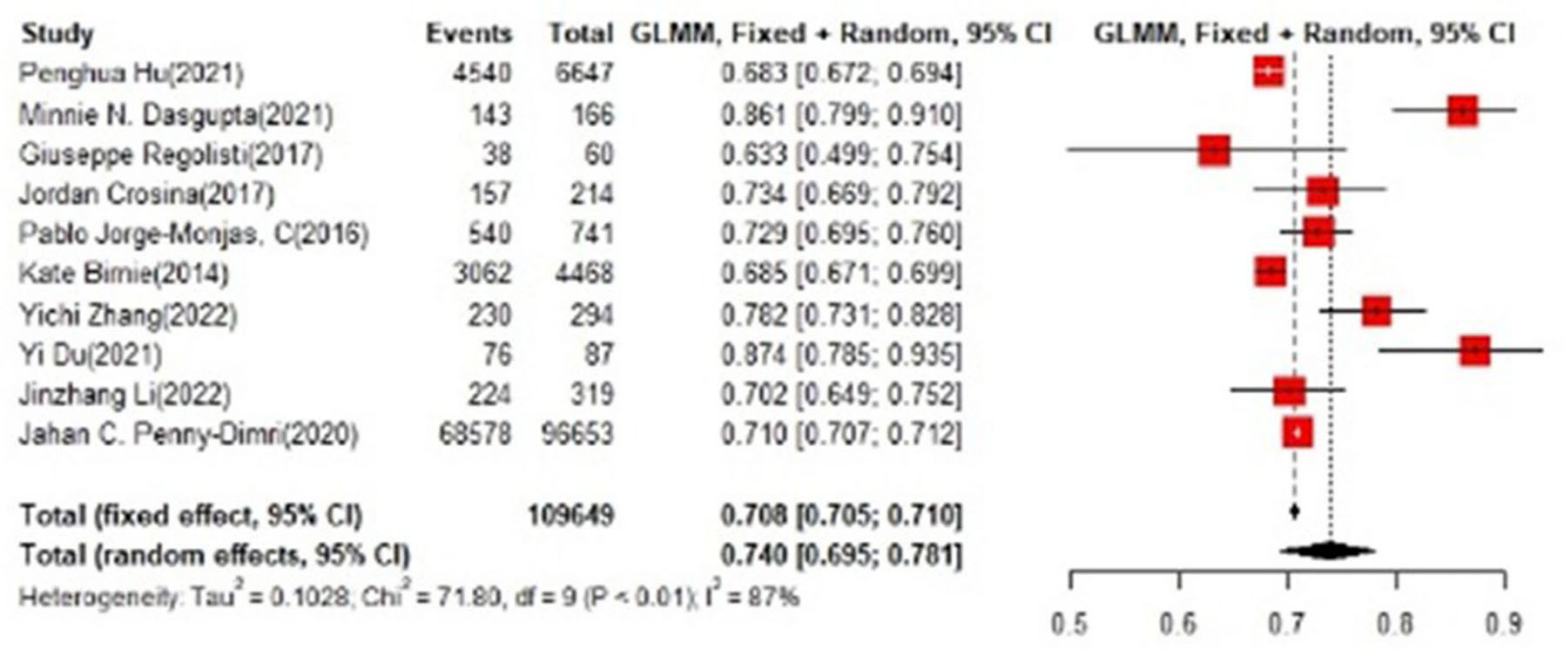
Accuracy of the machine learning-based model in the test set: logistic regression.

**Figure 6:**
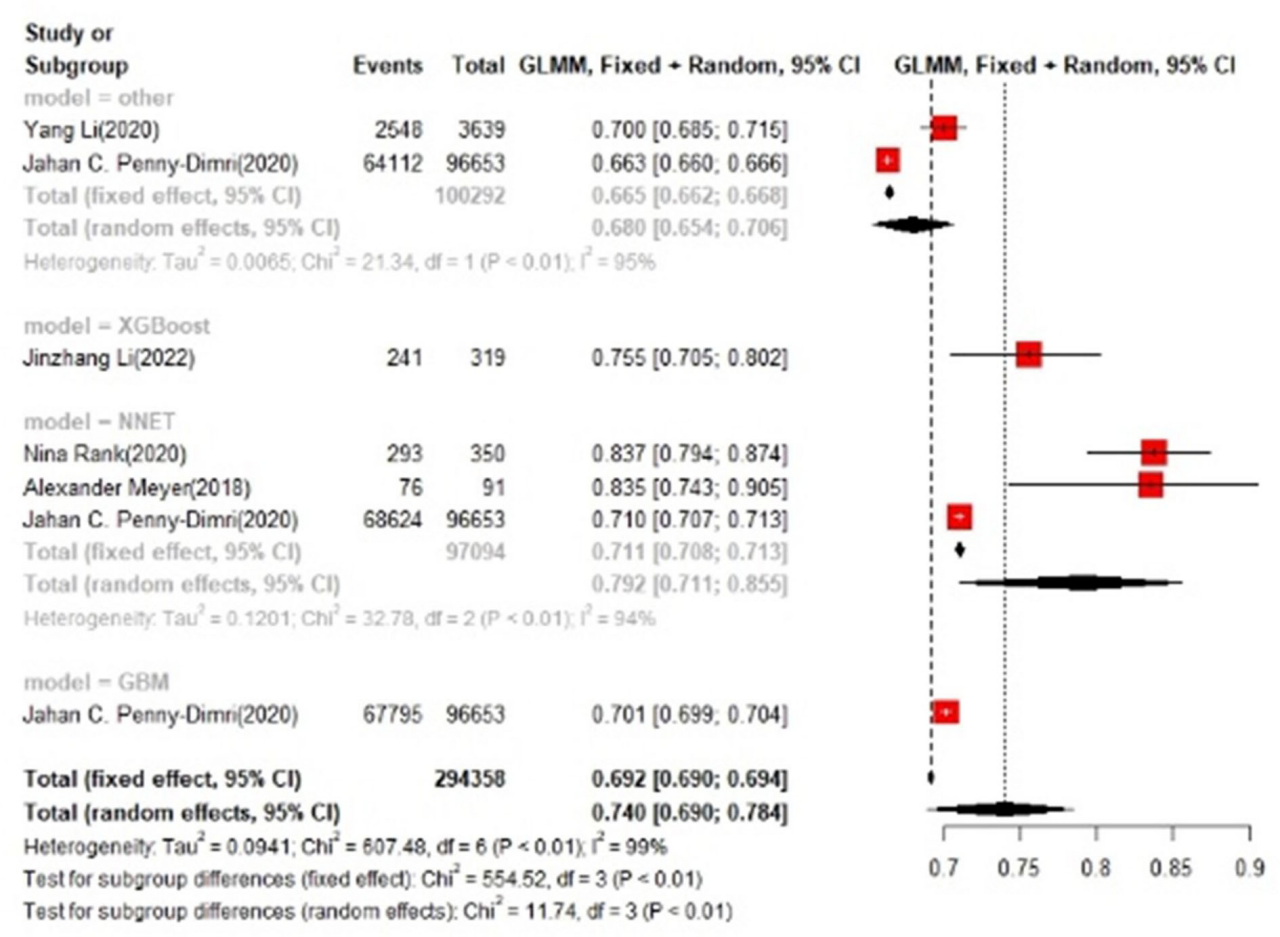
Accuracy of the machine learning-based model in the test set: other models.

In the test set, logistic regression was also the most commonly applied model [*n* = 10, accuracy = 0.708 (0.705, 0.71)]. NNET was of the best effect [*n* = 3, accuracy = 0.711 (0.708, 0.713)], with large modeling sample size, so we think NNET has the best effect. XGBoost also showed an excellent effect in all models [accuracy = 0.755 (0.705, 0.802)], while its modeling sample size was limited (Figures 5, 6).

### Training set and test set c-index

**Figure 7:**
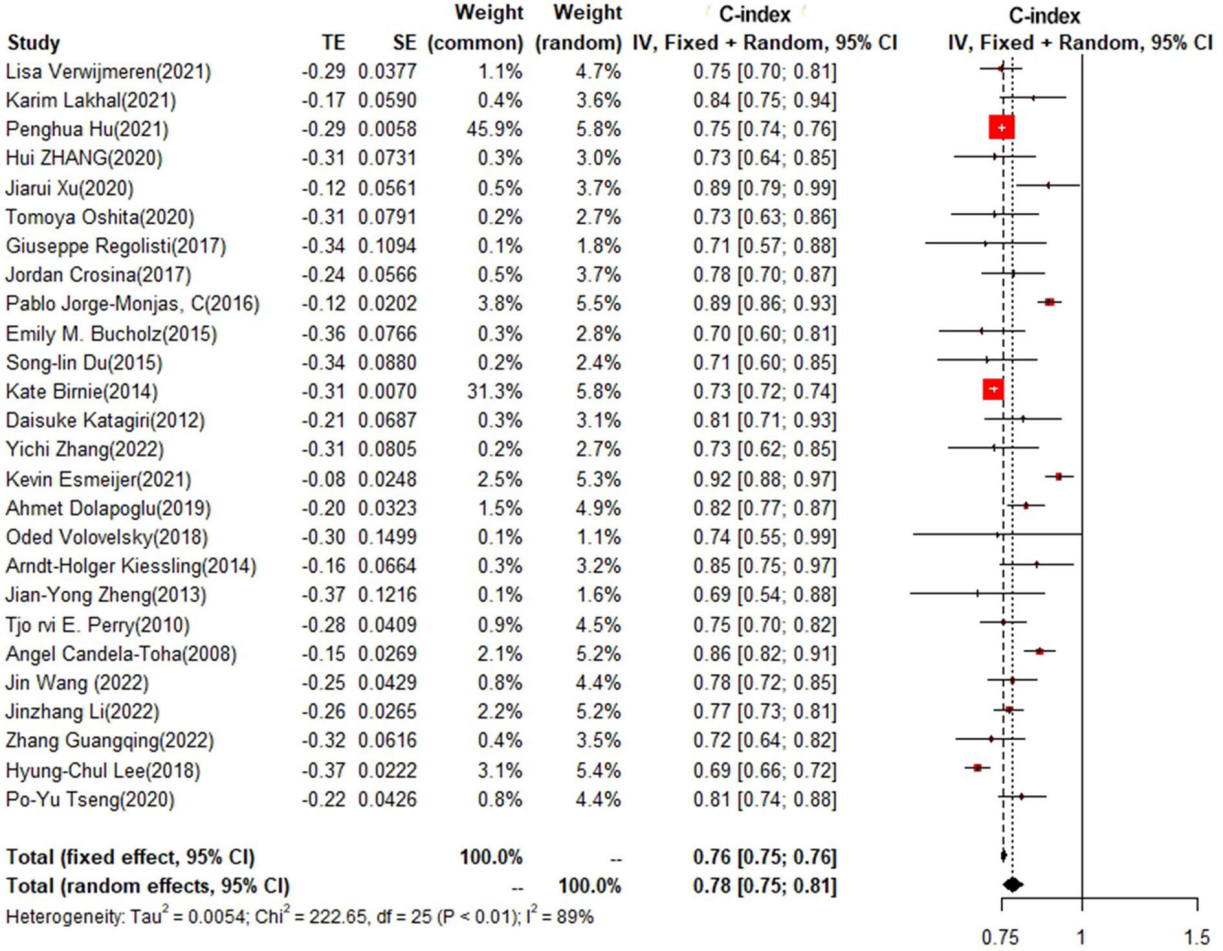
C-index in the training set: logistic regression.

**Figure 8:**
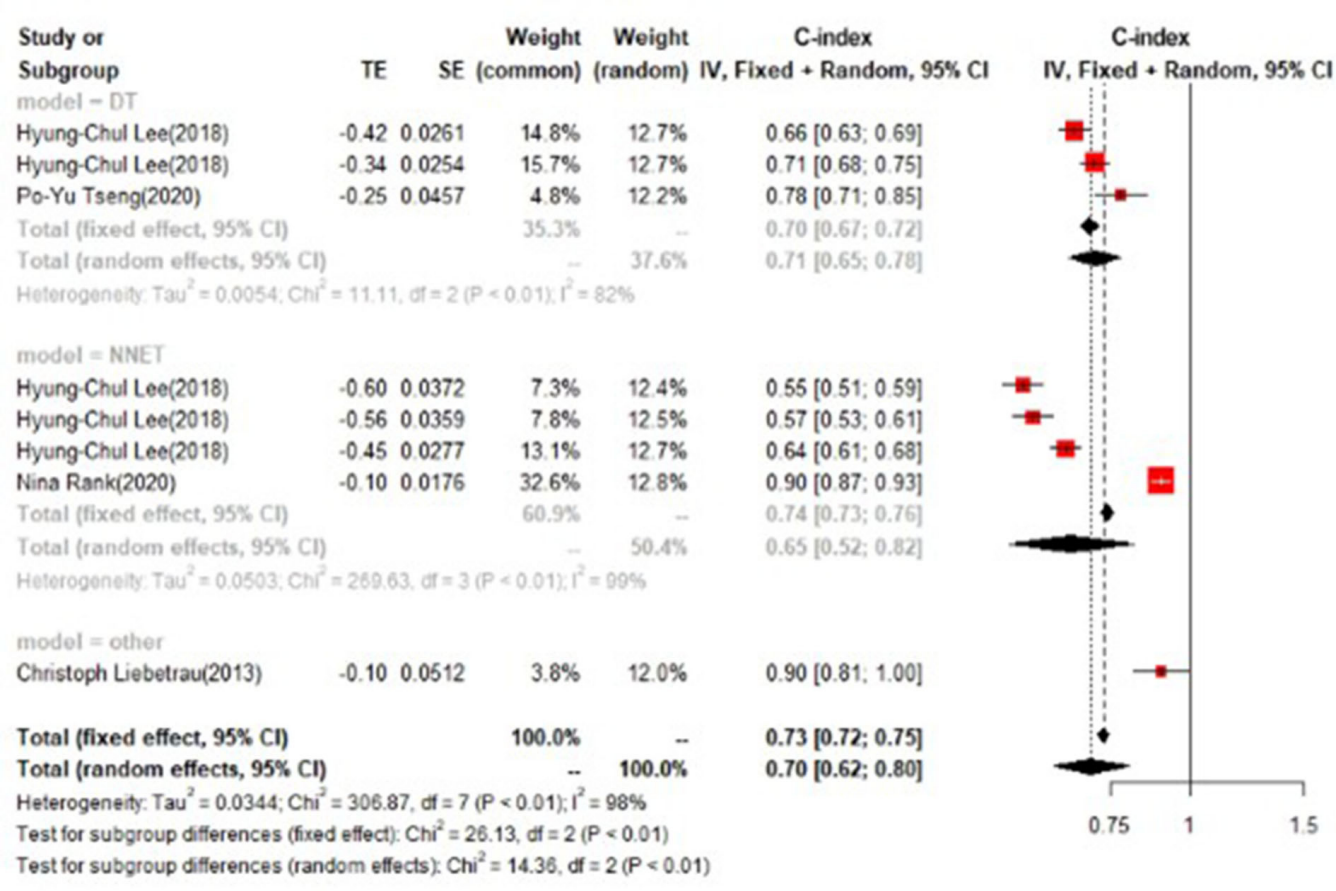
C-index in the training set: other models.

In the training set, logistic regression was the most commonly applied model [*n* = 26, c-index = 0.76 (0.75, 0.76)]. XGBoost showed to be of the best performance [*n* = 3, c-index =0.8 (0.78, 0.82)], with large modeling sample size. COX also showed a remarkable effect in all models [c-index = 0.9 (0.81, 1)], while its modeling sample size was limited (Figures 7, 8).

**Figure 9:**
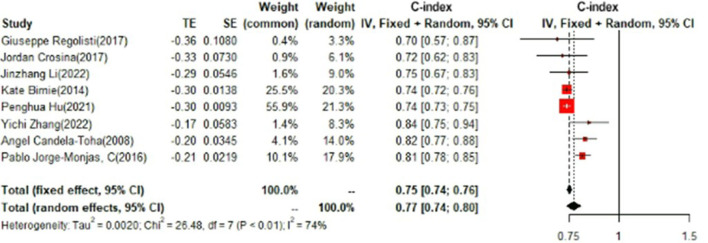
C-index in the test set: logistic regression.

**Figure 10:**
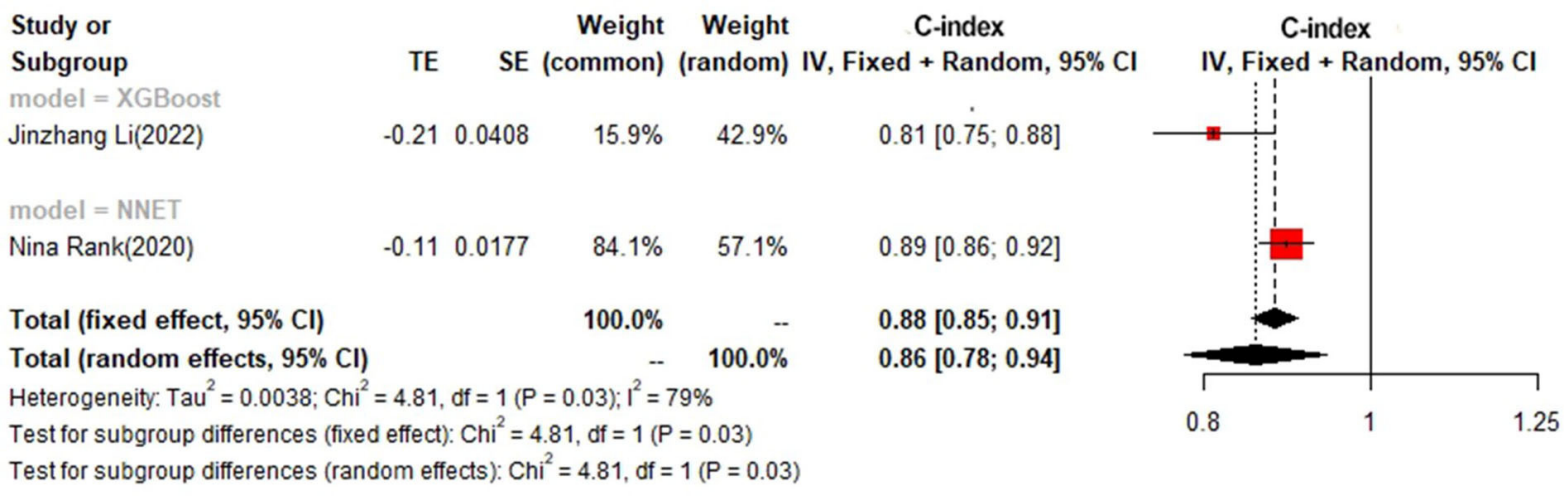
C-index in the test set: other models.

In the test set, logistic regression was also the most commonly applied model [*n* = 8, c-index = 0.75 (0.74, 0.76)]. NNET and XGBoost presented excellent performance, with the c-index of 0.89 (0.86, 0.92) and 0.81 (0.75, 0.88), respectively, while the modeling sample size of these two models was limited (Figures 9, 10).

Detailed results of data analyses are shown in Supplementary File 3.

## Discussion

There were 38 studies, with 60 CSA-AKI models, 12 external validity models, and 7 random sampling models. Most of them were logistic regression models. Risk of bias assessment showed that there was a high risk of bias in the analyses of included studies, which might be related to the incomplete variables and limited sample size involved in the model, improper processing of missing data, internal verification of models, interpretation of complex data, and correlation between some predictors and CSA-AKI definition. Therefore, follow-up studies should take into account the selection and verification of models, expansion of samples, and application of multivariate analysis. Data screening should also be more scientific to obtain more clinically valuable results.

Cardiac surgery altered the hemodynamics leading to hypoperfusion in the kidneys. Cardiac Angio Pulmonary Bypass can also induce hemodynamic disturbance directly and lead to acute kidney injury. Cardiac surgery-associated acute kidney injury (CSA-AKI) is a common and serious complication of cardiac surgery. There were more than 2 million people receiving cardiac surgery every year, and the incidence of CSA-AKI fluctuated between 5 and 42%. The occurrence of CSA-AKI is associated with high perioperative mortality, prolonged hospital stay, and heavy treatment costs. Pathogenesis of CSA-AKI should be further explored to elucidate the relationship between cardiac surgery undergoing and AKI occurring (2, 18).

Detailed mechanisms of CSA-AKI have not been fully elucidated. Ischemic reperfusion injury, activation of inflammatory cytokines cascades, oxidative stress, and nephrotoxic reaction might be involved in the pathogenesis of CSA-AKI (16, 19, 50). Hypotension may play a critical role in renal dysfunction, while the optimal mean arterial pressure (MAP) helpful to prevent CSA-AKI during CPB remains unknown. Almost all studies that assessed MAP during CPB were observational designs and were conducted to evaluate the correlation between hypotension and adverse neurologic outcomes. Griffin et al. (5) conducted a single-center RCT that included 300 patients who had known risk factors in AKI, and underwent elective cardiac surgery with normothermic CBP. They found that MAP during CPB was targeted to 50–60 mmHg in the control group, whereas that in the intervention group was targeted to 75–85 mmHg, and the overall mean MAP in the two groups was 60 ± 6 and 79 ± 6 mmHg, respectively. There was no intergroup difference in CSA-AKI, hospital LOS, and mortality.

AKI is a serious complication that can directly induce renal failure. The initiated injury process leads to irreversible renal function impairment that would continually deteriorate. Machine learning is capable of identifying the pathological factors of AKI so as to facilitate early intervention. Dong et al. (51) recruited 16,863 pediatric critical care patients aged from 1 month to 21 years, and used machine learning to predict pediatric AKI. Their findings were promising. Machine learning is a state-of-the-art approach to risk stratification. Interpretive modeling can use complex decision boundaries to help clinicians understand the risks specific to individual patients.

The application of machine learning is driven by the development of big data analysis and the need for evidence-based care. The practicality of the combination of artificial intelligence and machine learning has aroused widespread interest in medical research. Machine learning has its own advantages in performance and scalability, and machine learning-based modeling from mass data presents to be helpful to the implementation of dynamic monitoring for multiple diseases (52–55). Some machine learning algorithms, such as Extreme Gradient Boost (XGBoost), can calculate and predict the relative size of variables in a specific result, which makes the level of insight into individual risk factors and their prognostic significance comparable to that of logistic regression models (56). Gradient Boosting (GBM) is a widely used method to predict the incidence of AKI (57). Huang et al. (58) proposed a GBM-based risk prediction model for AKI after percutaneous coronary intervention (PCI). They collected a large amount of data from 947,091 patients receiving PCI to construct a baseline model, and time verification was carried out through the data of more than 900,000 hospitalized patients. The AUC of the GBM model was 79% larger than that of the baseline linear regression model. Lee et al. (59) proposed an AKI-prediction model based on several machine learning algorithms, and compared their performance in patients undergoing liver transplantation and heart surgery. Both the studies found that GBM had the most reliable performance.

In conclusion, CSA-AKI is a complex and multifaceted syndrome with significant morbidity and mortality. The application of machine learning in nephrotic clinical practice, including CSA-AKI, has a promising prospect.

We found that age, SCr, type of surgery, BMI, CBP time, and blood pressure were significant predictors for CSA-AKI. A large multinational and multicenter RCT, which involved 4,752 participants from 19 different countries, reported that Patients who underwent cardiac surgery without CPB were significantly less likely to have AKI 30 days after surgery [28 vs. 32.1%, RR = 0.87, 95% CI (80–0.96), *p* = 5.01] (17).

This systematic review and meta-analysis, based on a large sample size, showed that machine learning was effective in predicting the risk of CSA-AKI. Recently, the most common machine learning method is conventional logistic regression, followed by artificial neural networks, while SVM and RF are also commonly used. A study by Tseng et al. (16) demonstrated that machine learning could successfully predict CSA-AKI, which reflects the risks of cardiac surgery, enabling the optimization of postoperative treatment processes to diminish the postoperative complications following cardiac operations.

This study also has some limitations: first, this study focused on the accuracy of the machine learning model and did not predict the risk factors of CSA-AKI. Second, some of the included models contained special variables (such as SCr and eGFR), which were related to the diagnosis of AKI, and these variables would be of value for further verification and research in subsequent studies.

## Conclusion

Logistic regression remains the most commonly used model in CSA-AKI prediction, while it might not be the optimal option. Other models, such as NNET, XGBoost, and GBM, are of more remarkable performance. Using predictive models for early risk assessment has a relatively desirable effect on preventing CSA-AKI; however, it still needs to be further improved. Therefore, we look forward to more validated machine learning methods based on convenient, noninvasive, or minimally invasive predictive indicators that could be of remarkable performance and accuracy in the prediction of CSA-AKI.

## Data availability statement

The original contributions presented in the study are included in the article/Supplementary material, further inquiries can be directed to the corresponding author.

## Author contributions

ZS finished the entire research and bibliography retrieval. ZY was responsible for writing and review. MH and XS acted as the consultants. All authors contributed to the article and approved the submitted version.

## Funding

This study is supported entirely by the ZS. The whole study was finished without any external financial support.

## Conflict of interest

The authors declare that the research was conducted in the absence of any commercial or financial relationships that could be construed as a potential conflict of interest.

## Publisher's note

All claims expressed in this article are solely those of the authors and do not necessarily represent those of their affiliated organizations, or those of the publisher, the editors and the reviewers. Any product that may be evaluated in this article, or claim that may be made by its manufacturer, is not guaranteed or endorsed by the publisher.

## References

[1] Stevens PE Levin A Kidney Disease: Improving Global Outcomes Chronic Kidney Disease Guideline Development Work Group Members. Evaluation and management of chronic kidney disease: synopsis of the kidney disease: improving global outcomes 2012 clinical practice guideline. Ann Intern Med. (2013) 158:825–30. 10.7326/0003-4819-158-11-201306040-00007 23732715

[2] Massoth C Zarbock A Meersch M. Acute kidney injury in cardiac surgery. Crit Care Clin. (2021) 37:267–78. 10.1016/j.ccc.2020.11.009 33752855

[3] Wang Y Bellomo R. Cardiac surgery-associated acute kidney injury: risk factors, pathophysiology and treatment. Nat Rev Nephrol. (2017) 13:697–711. 10.1038/nrneph.2017.119 28869251

[4] Romagnoli S Ricci Z Ronco C. Perioperative acute kidney injury: prevention, early recognition, and supportive measures. Nephron. (2018) 140:105–10. 10.1159/000490500 29945154

[5] Griffin BR Liu KD Teixeira JP. Critical care nephrology: core curriculum 2020. Am J Kidney Dis. (2020) 75:435–52. 10.1053/j.ajkd.2019.10.010 31982214 PMC7333544

[6] Azau A Markowicz P Corbeau JJ Cottineau C Moreau X Baufreton C . Increasing mean arterial pressure during cardiac surgery does not reduce the rate of postoperative acute kidney injury. Perfusion. (2014) 29:496–504. 10.1177/0267659114527331 24619062

[7] Deo RC. Machine learning in medicine. Circulation. (2015) 132:1920–30. 10.1161/CIRCULATIONAHA.115.001593 26572668 PMC5831252

[8] Benjamin EJ Virani SS Callaway CW Chamberlain AM Chang AR Cheng S . Heart disease and stroke statistics-2018 update: a report from the American Heart Association. Circulation. (2018) 137:e67–e492. 10.1161/CIR.0000000000000558 29386200

[9] Vahanian A Beyersdorf F Praz F Milojevic M Baldus S Bauersachs J . 2021 ESC/EACTS Guidelines for the management of valvular heart disease: Developed by the Task Force for the management of valvular heart disease of the European Society of Cardiology (ESC) and the European Association for Cardio-Thoracic Surgery (EACTS). Rev Esp Cardiol. (2022) 75:524. 10.1016/j.rec.2022.05.006 35636831

[10] Moons KG de Groot JA Bouwmeester W Vergouwe Y Mallett S Altman DG . Critical appraisal and data extraction for systematic reviews of prediction modelling studies: the CHARMS checklist. PLoS Med. (2014) 11:e1001744. 10.1371/journal.pmed.1001744 25314315 PMC4196729

[11] Wolff RF Moons K Riley RD Whiting PF Westwood M Collins GS . PROBAST: a tool to assess the risk of bias and applicability of prediction model studies. Ann Intern Med. (2019) 170:51–8. 10.7326/M18-1376 30596875

[12] Verwijmeren L Bosma M Vernooij LM Linde EM Dijkstra IM Daeter EJ . Associations between preoperative biomarkers and cardiac surgery-associated acute kidney injury in elderly patients: a cohort study. Anesth Analg. (2021) 133:570–7. 10.1213/ANE.0000000000005650 34153017

[13] Kiessling AH Dietz J Reyher C Stock UA Beiras-Fernandez A Moritz A. Early postoperative serum cystatin C predicts severe acute kidney injury following cardiac surgery: a post-hoc analysis of a randomized controlled trial. J Cardiothorac Surg. (2014) 9:10. 10.1186/1749-8090-9-10 24397879 PMC3896845

[14] Guangqing Z Liwei C Fei L Jianshe Z Guang Z Yan Z . Predictive value of neutrophil to lymphocyte ratio on acute kidney injury after on-pump coronary artery bypass: a retrospective, single-center study. Gen Thorac Cardiovasc Surg. (2022) 70:624–33. 10.1007/s11748-022-01772-z 35103920 PMC9206599

[15] Lee HC Yoon HK Nam K Cho YJ Kim TK Kim WH . Derivation and validation of machine learning approaches to predict acute kidney injury after cardiac surgery. J Clin Med. (2018) 7:322. 10.3390/jcm7100322 30282956 PMC6210196

[16] Tseng PY Chen YT Wang CH Chiu KM Peng YS Hsu SP . Prediction of the development of acute kidney injury following cardiac surgery by machine learning. Crit Care. (2020) 24:478. 10.1186/s13054-020-03179-9 32736589 PMC7395374

[17] Rank N Pfahringer B Kempfert J Stamm C Kühne T Schoenrath F . Deep-learning-based real-time prediction of acute kidney injury outperforms human predictive performance. NPJ Digit Med. (2020) 3:139. 10.1038/s41746-020-00346-8 33134556 PMC7588492

[18] Meyer A Zverinski D Pfahringer B Kempfert J Kuehne T Sündermann SH . Machine learning for real-time prediction of complications in critical care: a retrospective study. Lancet Respir Med. (2018) 6:905–14. 10.1016/S2213-2600(18)30300-X 30274956

[19] Penny-Dimri JC Bergmeir C Reid CM Williams-Spence J Cochrane AD Smith JA. Machine learning algorithms for predicting and risk profiling of cardiac surgery-associated acute kidney injury. Semin Thorac Cardiovasc Surg. (2021) 33:735–45. 10.1053/j.semtcvs.2020.09.028 32979479

[20] Zhang H Zhou K Wang D Zhang N Liu J. The predictive value of the intraoperative Renal Pulsatility Index for acute kidney injury in patients undergoing cardiac surgery. Minerva Anestesiol. (2020) 86:1161–9. 10.23736/S0375-9393.20.14460-2 32615734

[21] Xu J Jiang W Li Y Shen B Shen Z Wang Y . Volume-associated hemodynamic variables for prediction of cardiac surgery-associated acute kidney injury. Clin Exp Nephrol. (2020) 24:798–805. 10.1007/s10157-020-01908-6 32494888

[22] Oshita T Hiraoka A Nakajima K Muraki R Arimichi M Chikazawa G . A better predictor of acute kidney injury after cardiac surgery: the largest area under the curve below the oxygen delivery threshold during cardiopulmonary bypass. J Am Heart Assoc. (2020) 9:e015566. 10.1161/JAHA.119.015566 32720572 PMC7792239

[23] Li Y Xu J Wang Y Zhang Y Jiang W Shen B . A novel machine learning algorithm, Bayesian networks model, to predict the high-risk patients with cardiac surgery-associated acute kidney injury. Clin Cardiol. (2020) 43:752–61. 10.1002/clc.23377 32400109 PMC7368305

[24] Zheng JY Xiao YY Yao Y Han L. Is serum cystatin C an early predictor for acute kidney injury following cardiopulmonary bypass surgery in infants and young children? Kaohsiung J Med Sci. (2013) 29:494–9. 10.1016/j.kjms.2013.01.004 24018153 PMC11916028

[25] Xu J Jiang W Li Y Li H Geng X Chen X . Association between syndecan-1, fluid overload, and progressive acute kidney injury after adult cardiac surgery. Front Med. (2021) 8:648397. 10.3389/fmed.2021.648397 34409046 PMC8366771

[26] Zivkovic N Elbaz-Greener G Qiu F Arbel Y Cheema AN Dvir D . Bedside risk score for prediction of acute kidney injury after transcatheter aortic valve replacement. Open heart. (2018) 5:e000777. 10.1136/openhrt-2018-000777 29862034 PMC5976119

[27] Regolisti G Maggiore U Cademartiri C Belli L Gherli T Cabassi A . Renal resistive index by transesophageal and transparietal echo-doppler imaging for the prediction of acute kidney injury in patients undergoing major heart surgery. J Nephrol. (2017) 30:243–53. 10.1007/s40620-016-0289-2 26995003

[28] Crosina J Lerner J Ho J Tangri N Komenda P Hiebert B . Improving the prediction of cardiac surgery-associated acute kidney injury. Kidney Int Rep. (2016) 2:172–9. 10.1016/j.ekir.2016.10.003 29142955 PMC5678656

[29] Jorge-Monjas P Bustamante-Munguira J Lorenzo M Heredia-Rodríguez M Fierro I Gómez-Sánchez E . Predicting cardiac surgery-associated acute kidney injury: The CRATE score. J Crit Care. (2016) 31:130–8. 10.1016/j.jcrc.2015.11.004 26700607

[30] Heringlake M Charitos EI Erber K Berggreen AE Heinze H Paarmann H. Preoperative plasma growth-differentiation factor-15 for prediction of acute kidney injury in patients undergoing cardiac surgery. Crit Care. (2016) 20:317. 10.1186/s13054-016-1482-3 27717384 PMC5055664

[31] Shah KS Taub P Patel M Rehfeldt M Struck J Clopton P . Proenkephalin predicts acute kidney injury in cardiac surgery patients. Clin Nephrol. (2015) 83:29–35. 10.5414/CN108387 25512100

[32] Bucholz EM Whitlock RP Zappitelli M Devarajan P Eikelboom J Garg AX . Cardiac biomarkers and acute kidney injury after cardiac surgery. Pediatrics. (2015) 135:e945–56. 10.1542/peds.2014-2949 25755241 PMC4379461

[33] Du SL Zeng XZ Tian JW Ai J Wan J He JX. Advanced oxidation protein products in predicting acute kidney injury following cardiac surgery. Biomarkers. (2015) 20:206–11. 10.3109/1354750X.2015.1062917 26154394

[34] Birnie K Verheyden V Pagano D Bhabra M Tilling K Sterne JA . Predictive models for kidney disease: improving global outcomes (KDIGO) defined acute kidney injury in UK cardiac surgery. Crit Care. (2014) 18:606. 10.1186/s13054-014-0606-x 25673427 PMC4258283

[35] Hu P Mo Z Chen Y Wu Y Song L Zhang L . Derivation and validation of a model to predict acute kidney injury following cardiac surgery in patients with normal renal function. Ren Fail. (2021) 43:1205–13. 10.1080/0886022X.2021.1960563 34372744 PMC8354173

[36] Liebetrau C Dörr O Baumgarten H Gaede L Szardien S Blumenstein J . Neutrophil gelatinase-associated lipocalin (NGAL) for the early detection of cardiac surgery associated acute kidney injury. Scand J Clin Lab Invest. (2013) 73:392–9. 10.3109/00365513.2013.787149 23668886

[37] Katagiri D Doi K Honda K Negishi K Fujita T Hisagi M . Combination of two urinary biomarkers predicts acute kidney injury after adult cardiac surgery. Ann Thorac Surg. (2012) 93:577–83. 10.1016/j.athoracsur.2011.10.048 22269724

[38] Zhang Y Zhao H Su Q Wang C Chen H Shen L . Novel plasma biomarker-based model for predicting acute kidney injury after cardiac surgery: a case control study. Front Med. (2022) 8:799516. 10.3389/fmed.2021.799516 35096889 PMC8795513

[39] Esmeijer K Schoe A Ruhaak LR Hoogeveen EK Soonawala D Romijn F . The predictive value of TIMP-2 and IGFBP7 for kidney failure and 30-day mortality after elective cardiac surgery. Sci Rep. (2021) 11:1071. 10.1038/s41598-020-80196-2 33441876 PMC7806984

[40] Du Y Wang XZ Wu WD Shi HP Yang XJ Wu WJ . Predicting the risk of acute kidney injury in patients after percutaneous coronary intervention (PCI) or cardiopulmonary bypass (CPB) surgery: development and assessment of a nomogram prediction model. Med Sci Monit. (2021) 27:e929791. 10.12659/MSM.929791 33895770 PMC8083792

[41] Dolapoglu A Avci E Kiris T Bugra O. The predictive value of the prognostic nutritional index for postoperative acute kidney injury in patients undergoing on-pump coronary bypass surgery. J Cardiothorac Surg. (2019) 14:74. 10.1186/s13019-019-0898-7 30971264 PMC6458745

[42] Lakhal K Bigot-Corbel E Sacchetto E Chabrun F Senage T Figueres L . Early recognition of cardiac surgery-associated acute kidney injury: (an observational pilot study). BMC Anesthesiol. (2021) 21:244. 10.1186/s12871-021-01387-6 34641779 PMC8513334

[43] Volovelsky O Gist KM Terrell TC Bennett MR Cooper DS Alten JA . Early postoperative measurement of fibroblast growth factor 23 predicts severe acute kidney injury in infants after cardiac surgery. Clin Nephrol. (2018) 90:165–71. 10.5414/CN109359 29633705 PMC6350240

[44] Perry TE Muehlschlegel JD Liu KY Fox AA Collard CD Shernan SK . Plasma neutrophil gelatinase-associated lipocalin and acute postoperative kidney injury in adult cardiac surgical patients. Anesth Analg. (2010) 110:1541–7. 10.1213/ANE.0b013e3181da938e 20435938 PMC2999841

[45] Dasgupta MN Montez-Rath ME Hollander SA Sutherland SM. Using kinetic eGFR to identify acute kidney injury risk in children undergoing cardiac transplantation. Pediatr Res. (2021) 90:632–6. 10.1038/s41390-020-01307-3 33446916

[46] Portilla D Dent C Sugaya T Nagothu KK Kundi I Moore P . Liver fatty acid-binding protein as a biomarker of acute kidney injury after cardiac surgery. Kidney Int. (2008) 73:465–72. 10.1038/sj.ki.5002721 18094680

[47] Candela-Toha A Elías-Martín E Abraira V Tenorio MT Parise D de Pablo A . Predicting acute renal failure after cardiac surgery: external validation of two new clinical scores. Clin J Am Soc Nephrol. (2008) 3:1260–5. 10.2215/CJN.00560208 18463173 PMC2518781

[48] Wang J Yu C Zhang Y Huang Y. A prediction model for acute kidney injury after pericardiectomy: an observational study. Front Cardiovasc Med. (2022) 9:790044. 10.3389/fcvm.2022.790044 35224038 PMC8873385

[49] Li J Gong M Joshi Y Sun L Huang L Fan R . Machine learning prediction model for acute renal failure after acute aortic syndrome surgery. Front Med. (2022) 8:728521. 10.3389/fmed.2021.728521 35111767 PMC8801502

[50] Sutherland L Hittesdorf E Yoh N Lai T Mechling A Wagener G. Acute kidney injury after cardiac surgery: a comparison of different definitions. Nephrology. (2020) 25:212–8. 10.1111/nep.13669 31587419

[51] Dong J Feng T Thapa-Chhetry B Cho BG Shum T Inwald DP . Machine learning model for early prediction of acute kidney injury (AKI) in pediatric critical care. Crit Care. (2021) 25:288. 10.1186/s13054-021-03724-0 34376222 PMC8353807

[52] Shorten G Srinivasan KK Reinertsen I. Machine learning and evidence-based training in technical skills. Br J Anaesth. (2018) 121:521–3. 10.1016/j.bja.2018.04.012 30115245

[53] Ghahramani Z. Probabilistic machine learning and artificial intelligence. Nature. (2015) 521:452–9. 10.1038/nature14541 26017444

[54] Quax S van Gerven M. Emergent mechanisms of evidence integration in recurrent neural networks. PLoS ONE. (2018) 13:e0205676. 10.1371/journal.pone.0205676 30325970 PMC6191121

[55] Abraham A Pedregosa F Eickenberg M Gervais P Mueller A Kossaifi J . Machine learning for neuroimaging with scikit-learn. Front Neuroinf . (2014) 8:14. 10.3389/fninf.2014.00014 24600388 PMC3930868

[56] Kilic A. Artificial Intelligence and Machine Learning in Cardiovascular Health Care. Ann Thorac Surg. (2020) 109:1323–9. 10.1016/j.athoracsur.2019.09.042 31706869

[57] Koyner JL Carey KA Edelson DP Churpek MM. The development of a machine learning inpatient acute kidney injury prediction model. Crit Care Med. (2018) 46:1070–7. 10.1097/CCM.0000000000003123 29596073

[58] Huang C Murugiah K Mahajan S Li SX Dhruva SS Haimovich JS . Enhancing the prediction of acute kidney injury risk after percutaneous coronary intervention using machine learning techniques: a retrospective cohort study. PLoS Med. (2018) 15:e1002703. 10.1371/journal.pmed.1002703 30481186 PMC6258473

[59] Lee HC Yoon SB Yang SM Kim WH Ryu HG Jung CW . Prediction of acute kidney injury after liver transplantation: machine learning approaches vs. logistic regression model. J Clin Med. (2018) 7:428. 10.3390/jcm7110428 30413107 PMC6262324

