# Supplementary material for: Machine learning in predicting cardiac surgery-associated acute kidney injury: A systemic review and meta-analysis
Source: Front Cardiovasc Med. 2022 Sep 15;9:951881. doi: 10.3389/fcvm.2022.951881 (PMC9520338; doi:10.3389/fcvm.2022.951881)
Supplement: Supplementary file 1 [file Table_1.docx]

**Supplementary File 1：****Basic information of enrolled articles**

| **study** | **country** | **type of study** | **missing data** | **number of AKI samples in training set** | **total number of samples in training set** | **verification set generation method** | **number of AKI samples in test set** | **total number of samples in test set** | **model type** | **number of model variables** |
| --- | --- | --- | --- | --- | --- | --- | --- | --- | --- | --- |
| Lisa Verwijmeren(2021) | Netherlands | Prospectively | multiple imputation | 88 | 539 | -- | -- | -- | LR | 4 |
| Jiarui Xu(2021) | China | Prospectively | -- | 43 | 167 | -- | -- | -- | LR | 2 |
| Karim Lakhal(2021) | France | Prospectively | -- | 27 | 65 | -- | -- | -- | LR | 5 |
| Penghua Hu(2021) | China | retrospective cohort study | multiple imputation | 3955 | 15701 | random sampling | 1621 | 6647 | LR | 9 |
| MinnieN. Dasgupta(2021) | USA | retrospective cohort study | -- | 131 | 175 | multicenter | 124 | 166 | LR | 2 |
| Hui ZHANG(2020) | China | Prospectively | multiple imputation | 23 | 78 | -- | -- | -- | LR | 4 |
| Jiarui Xu(2020) | China | retrospective cohort study | -- | 433 | 1180 | -- | -- | -- | LR | 7 |
| Tomoya Oshita(2020) | Japan | retrospective case-controlled study | multiple imputation | 25 | 202 | -- | -- | -- | LR | 3 |
| Yang Li(2020) | China | retrospective case-controlled study | multiple imputation | 1364 | 5533 | multicenter | 405 | 3639 | BNs | 13 |
| Nevena Zivkovic(2018) | Canada | retrospective cohort study | multiple imputation | 318 | 1993 | -- | -- | -- | LR | 6 |
| Giuseppe Regolisti(2017) | Italy | Prospectively | -- | 23 | 60 | multicenter | 23 | 60 | LR | 11 |
| Jordan Crosina(2017) | Canada | Prospectively | multiple imputation | 35 | 289 | multicenter | 16 | 214 | LR | 5 |
| Pablo Jorge-Monjas, C(2016) | Spain | retrospective cohort study | multiple imputation | 137 | 810 | multicenter | 117 | 741 | LR | 4 |
| Matthias Heringlake(2016) | USA | Prospectively | multiple imputation | 258 | 1176 | -- | -- | -- | LR、RF | 15 |
| Kevin S. Shah(2015) | USA | Prospectively | -- | 20 | 92 | -- | -- | -- | LR | 2 |
| Emily M. Bucholz(2015) | Canada | Prospectively | multiple imputation | 55 | 106 | -- | -- | -- | LR | 5 |
| Song-lin Du(2015) | China | Prospectively | multiple imputation | 35 | 67 | -- | -- | -- | LR | 8 |
| Kate Birnie(2014) | UK | Prospectively | multiple imputation | 855 | 4092 | multicenter | 1099 | 4468 | LR | 5 |
| Christoph Liebetrau(2013) | Germany | retrospective cohort study | -- | 47 | 141 | -- | -- | -- | COX | 6 |
| Daisuke Katagiri(2012) | Japan | Prospectively | -- | 28 | 77 | -- | -- | -- | LR | 3 |
| Yichi Zhang(2022) | China | retrospective case-controlled study | multiple imputation | 17 | 131 | random sampling | 50 | 294 | NNET | 7 |
| Kevin Esmeijer(2021) | The Netherlands | retrospective cohort study | multiple imputation | 22 | 344 | -- | -- | -- | LR | 3 |
| Yi Du(2021) | China | Prospectively | multiple imputation | 40 | 204 | random sampling | 18 | 87 | RF、LR | 10 |
| Ahmet Dolapoglu(2019) | Turkey | retrospective cohort study | multiple imputation | 88 | 336 | -- | -- | -- | LR | 4 |
| Oded Volovelsky(2018) | USA | Prospectively | multiple imputation | 9 | 41 | -- | -- | -- | LR | 3 |
| Arndt-Holger Kiessling(2014) | Germany | Prospectively | multiple imputation | 75 | 139 | -- | -- | -- | LR | 2 |
| Jian-Yong Zheng(2013) | China | Prospectively | -- | 21 | 43 | -- | -- | -- | LR | 4 |
| Tjo rvi E. Perry(2010) | USA | retrospective observational study | -- | 879 | 1141 | -- | -- | -- | LR | 7 |
| D Portilla(2008) | USA | retrospective cohort study | -- | 21 | 40 | -- | -- | -- | LR | 5 |
| Angel Candela-Toha(2008) | Spain | retrospective cohort study | multiple imputation | 67 | 1780 | multicenter | 58 | 1563 | LR | 13 |
| Jin Wang (2022) | China | retrospective cohort study | multiple imputation | 95 | 211 | -- | -- | -- | LR | 8 |
| Jinzhang Li(2022) | China | retrospective cohort study | k-nearest neighbors approach | 151 | 1318 | random sampling | 35 | 319 | XGBoost、LR、RF | 5 |
| Zhang Guangqing(2022) | China | retrospective, single‑center study | -- | 57 | 154 | -- | -- | -- | LR | 2 |
| Hyung-Chul Lee(2018) | Korea | retrospective observational study | multiple imputation | 375 | 1005 | multicenter | 395 | 1005 | DT、RF、XGBoost、NNET | 11 |
| Po-Yu Tseng(2020) | China | retrospective observational study | -- | 114 | 468 | random sampling | 49 | 202 | LR、SVM、RF、XGBoost | -- |
| Nina Rank(2020) | Germany | retrospective observational study | multiple imputation | 1487 | 2080 | random sampling | 245 | 350 | NNET | 96 |
| Alexander Meyer(2018) | Germany | retrospective observational study | multiple imputation | 407 | 813 | multicenter | 45 | 91 | NNET | 52 |
| Jahan C. Penny-Dimri(2020) | Australia | Prospectively | multiple imputation | 4599 | 96653 | multicenter | 4599 | 96653 | LR、GBM、NNET | 56 |
