# Supplementary material for: Machine learning in predicting cardiac surgery-associated acute kidney injury: A systemic review and meta-analysis
Source: Front Cardiovasc Med. 2022 Sep 15;9:951881. doi: 10.3389/fcvm.2022.951881 (PMC9520338; doi:10.3389/fcvm.2022.951881)
Supplement: Supplementary file 2 [file Table_2.docx]

**Supplementary File 2：searching constructions**

| Database | Retrieval formular | | Result |
| --- | --- | --- | --- |
| Cochrane | #1 | MeSH descriptor: [Thoracic Surgery] explode all trees | 174 |
|  | #2 | (Surgery, Thoracic):ti,ab,kw OR (Surgery, Cardiac):ti,ab,kw OR (Surgery, Heart):ti,ab,kw OR (Heart Surgery):ti,ab,kw AND (Cardiac Surgery):ti,ab,kw | 42223 |
|  | #3 | MeSH descriptor: [Machine Learning] explode all trees | 200 |
|  | #4 | (machine learning):ti,ab,kw OR (Deep learning):ti,ab,kw OR (Prediction model):ti,ab,kw OR (Transfer Learning):ti,ab,kw OR (random forest):ti,ab,kw | 8468 |
|  | #5 | (artificial neural network):ti,ab,kw OR (ANN):ti,ab,kw OR (Support vector machine):ti,ab,kw OR (SVM):ti,ab,kw OR (Nomogram):ti,ab,kw | 3599 |
|  | #6 | (COX):ti,ab,kw OR (XGboost):ti,ab,kw OR (Logistic):ti,ab,kw OR (Decision tree):ti,ab,kw OR (c-index):ti,ab,kw | 46211 |
|  | #7 | (ROC):ti,ab,kw OR (AUC):ti,ab,kw OR (External validation):ti,ab,kw | 24634 |
|  | #8 | #1 or #2 | 42223 |
|  | #9 | #3 or #4 or #5 or #6 or #7 | 76484 |
|  | #10 | MeSH descriptor: [Acute Kidney Injury] explode all trees | 1518 |
|  | #11 | (Acute Kidney Injuries):ti,ab,kw OR (Kidney Injuries, Acute):ti,ab,kw OR (Kidney Injury, Acute):ti,ab,kw OR (Acute Renal Injury):ti,ab,kw OR (Acute Renal Injuries):ti,ab,kw | 4894 |
|  | #12 | (Renal Injuries, Acute):ti,ab,kw OR (Renal Injury, Acute):ti,ab,kw OR (Renal Insufficiency, Acute):ti,ab,kw OR (Acute Renal Insufficiencies):ti,ab,kw OR (Renal Insufficiencies, Acute):ti,ab,kw | 3886 |
|  | #13 | (Acute Renal Insufficiency):ti,ab,kw OR (Kidney Insufficiency, Acute):ti,ab,kw OR (Acute Kidney Insufficiencies):ti,ab,kw OR (Kidney Insufficiencies, Acute):ti,ab,kw OR (Acute Kidney Insufficiency):ti,ab,kw | 1158 |
|  | #14 | (Kidney Failure, Acute):ti,ab,kw OR (Acute Kidney Failures):ti,ab,kw OR (Kidney Failures, Acute):ti,ab,kw OR (Acute Renal Failure):ti,ab,kw OR (Acute Renal Failures):ti,ab,kw | 8230 |
|  | #15 | (Renal Failures, Acute):ti,ab,kw OR (Renal Failure, Acute):ti,ab,kw OR (Acute Kidney Failure):ti,ab,kw | 8197 |
|  | #16 | #10 or #11 or #12 or #13 or #14 or #15 | 10425 |
|  | #17 | #8 and #9 and #16 | 133 |
| Web of science | #1 | Thoracic Surgery (Topic) or Surgery, Thoracic (Topic) or Surgery, Cardiac (Topic) or Surgery, Heart (Topic) or Heart Surgery (Topic) or Cardiac Surgery (Topic) | 185361 |
|  | #2 | machine learning (Topic) or machine learning (Topic) or Deep learning (Topic) or Prediction model (Topic) or Transfer Learning (Topic) or random forest (Topic) or artificial neural network (Topic) or ANN (Topic) or Support vector machine (Topic) or SVM (Topic) or Nomogram (Topic) or COX (Topic) or XGboost (Topic) or Logistic (Topic) or Decision tree (Topic) or c-index (Topic) or ROC (Topic) or AUC (Topic) or External validation (Topic) | 2168602 |
|  | #3 | Acute Kidney Injury (Topic) or Acute Kidney Injuries (Topic) or Kidney Injuries, Acute (Topic) or Kidney Injury, Acute (Topic) or Acute Renal Injury (Topic) or Acute Renal Injuries (Topic) or Renal Injuries, Acute (Topic) or Renal Injury, Acute (Topic) or Renal Insufficiency, Acute (Topic) or Acute Renal Insufficiencies (Topic) or Renal Insufficiencies, Acute (Topic) or Acute Renal Insufficiency (Topic) or Kidney Insufficiency, Acute (Topic) or Acute Kidney Insufficiencies (Topic) or Kidney Insufficiencies, Acute (Topic) or Acute Kidney Insufficiency (Topic) or Kidney Failure, Acute (Topic) or Acute Kidney Failures (Topic) or Kidney Failures, Acute (Topic) or Acute Renal Failure (Topic) or Acute Renal Failures (Topic) or Renal Failures, Acute (Topic) or Renal Failure, Acute (Topic) or Acute Kidney Failure (Topic) | 91833 |
|  | #4 | #1 AND #2 AND #3 | 1512 |
| Embase | #1 | 'thoracic surgery':ti,ab,kw OR 'surgery, thoracic':ti,ab,kw OR 'surgery, cardiac':ti,ab,kw OR 'surgery, heart':ti,ab,kw OR 'heart surgery':ti,ab,kw OR 'cardiac surgery':ti,ab,kw | 116249 |
|  | #2 | 'machine learning':ti,ab,kw OR 'deep learning':ti,ab,kw OR 'prediction model':ti,ab,kw OR 'transfer learning':ti,ab,kw OR 'random forest':ti,ab,kw OR 'artificial neural network':ti,ab,kw OR ann:ti,ab,kw OR 'support vector machine':ti,ab,kw OR svm:ti,ab,kw OR nomogram:ti,ab,kw OR cox:ti,ab,kw OR xgboost:ti,ab,kw OR logistic:ti,ab,kw OR 'decision tree':ti,ab,kw OR 'c index':ti,ab,kw OR roc:ti,ab,kw OR auc:ti,ab,kw OR 'external validation':ti,ab,kw | 1262600 |
|  | #3 | 'acute kidney injury network criteria':ti,ab,kw OR 'acute kidney injuries':ti,ab,kw OR 'kidney injuries, acute':ti,ab,kw OR 'kidney injury, acute':ti,ab,kw OR 'acute renal injury':ti,ab,kw OR 'acute renal injuries':ti,ab,kw OR 'renal injuries, acute':ti,ab,kw OR 'renal injury, acute':ti,ab,kw OR 'renal insufficiency, acute':ti,ab,kw OR 'acute renal insufficiencies':ti,ab,kw OR 'renal insufficiencies, acute':ti,ab,kw OR 'acute renal insufficiency':ti,ab,kw OR 'acute kidney insufficiencies':ti,ab,kw OR 'kidney insufficiencies, acute':ti,ab,kw OR 'kidney failure, acute':ti,ab,kw OR 'acute kidney failures':ti,ab,kw OR 'kidney failures, acute':ti,ab,kw OR 'acute renal failure':ti,ab,kw OR 'acute renal failures':ti,ab,kw OR 'renal failures, acute':ti,ab,kw OR 'renal failure, acute':ti,ab,kw OR 'acute kidney failure':ti,ab,kw | 41419 |
|  | #4 | #1 AND #2 AND #3 | 231 |
| PubMed | #1 | (((((Thoracic Surgery[Title/Abstract]) OR (Surgery, Thoracic[Title/Abstract])) OR (Surgery, Cardiac[Title/Abstract])) OR (Surgery, Heart[Title/Abstract])) OR (Heart Surgery[Title/Abstract])) OR (Cardiac Surgery[Title/Abstract]) | 77527 |
|  | #2 | ((((((((((((((((((machine learning[Title/Abstract]) OR (machine learning[Title/Abstract])) OR (Deep learning[Title/Abstract])) OR (Prediction model[Title/Abstract])) OR (Transfer Learning[Title/Abstract])) OR (random forest[Title/Abstract])) OR (artificial neural network[Title/Abstract])) OR (ANN[Title/Abstract])) OR (Support vector machine[Title/Abstract])) OR (SVM[Title/Abstract])) OR (Nomogram[Title/Abstract])) OR (COX[Title/Abstract])) OR (XGboost[Title/Abstract])) OR (Logistic[Title/Abstract])) OR (Decision tree[Title/Abstract])) OR (c-index[Title/Abstract])) OR (ROC[Title/Abstract])) OR (AUC[Title/Abstract])) OR (External validation[Title/Abstract]) | 817360 |
|  | #3 | (((((((((((((((((((((((Acute Kidney Injury[Title/Abstract]) OR (Acute Kidney Injuries[Title/Abstract])) OR (Kidney Injuries, Acute[Title/Abstract])) OR (Kidney Injury, Acute[Title/Abstract])) OR (Acute Renal Injury[Title/Abstract])) OR (Acute Renal Injuries[Title/Abstract])) OR (Renal Injuries, Acute[Title/Abstract])) OR (Renal Injury, Acute[Title/Abstract])) OR (Renal Insufficiency, Acute[Title/Abstract])) OR (Acute Renal Insufficiencies[Title/Abstract])) OR (Renal Insufficiencies, Acute[Title/Abstract])) OR (Acute Renal Insufficiency[Title/Abstract])) OR (Kidney Insufficiency, Acute[Title/Abstract])) OR (Acute Kidney Insufficiencies[Title/Abstract])) OR (Kidney Insufficiencies, Acute[Title/Abstract])) OR (Acute Kidney Insufficiency[Title/Abstract])) OR (Kidney Failure, Acute[Title/Abstract])) OR (Acute Kidney Failures[Title/Abstract])) OR (Kidney Failures, Acute[Title/Abstract])) OR (Acute Renal Failure[Title/Abstract])) OR (Acute Renal Failures[Title/Abstract])) OR (Renal Failures, Acute[Title/Abstract])) OR (Renal Failure, Acute[Title/Abstract])) OR (Acute Kidney Failure[Title/Abstract]) | 71673 |
|  | #4 | #1 AND #2 AND #3 | 33 |
