# Supplementary material for: Machine learning in predicting cardiac surgery-associated acute kidney injury: A systemic review and meta-analysis
Source: Front Cardiovasc Med. 2022 Sep 15;9:951881. doi: 10.3389/fcvm.2022.951881 (PMC9520338; doi:10.3389/fcvm.2022.951881)
Supplement: Supplementary file 3 [file Table_3.DOCX]

| Outcome | Model | Training | | | | | Test | | | | |
| --- | --- | --- | --- | --- | --- | --- | --- | --- | --- | --- | --- |
|  |  | n | Total | ACC | low | up | n | Total | ACC | low | up |
| accuracy | LR | 27 | 125949 | 0.705 | 0.703 | 0.708 | 10 | 109649 | 0.708 | 0.705 | 0.71 |
|  | DT | 3 | 2478 | 0.655 | 0.636 | 0.674 | - | - | - | - | - |
|  | SVM | 4 | 3483 | 0.641 | 0.625 | 0.657 | - | - | - | - | - |
|  | XGBoost | 3 | 2791 | 0.732 | 0.715 | 0.748 | 1 | 319 | 0.755 | 0.705 | 0.802 |
|  | NNET | 6 | 102561 | 0.710 | 0.707 | 0.713 | 3 | 97094 | 0.711 | 0.708 | 0.713 |
|  | RF | 4 | 2946 | 0.667 | 0.649 | 0.683 | - | - | - | - | - |
|  | GBM | 1 | 67795 | 0.701 | 0.699 | 0.704 | 1 | 96653 | 0.701 | 0.699 | 0.704 |
|  | other | 3 | 102327 | 0.666 | 0.663 | 0.668 | 2 | 100292 | 0.665 | 0.662 | 0.668 |
|  | Overall | 51 | 410330 | 0.720 | 0.700 | 0.730 | 17 | 404007 | 0.73 | 0.71 | 0.74 |
| C-index | LR | 26 | 30377 | 0.760 | 0.750 | 0.760 | 8 | 14306 | 0.75 | 0.74 | 0.76 |
|  | DT | 3 | 2478 | 0.700 | 0.670 | 0.720 | - | - | - | - | - |
|  | SVM | 4 | 3483 | 0.690 | 0.680 | 0.710 | - | - | - | - | - |
|  | XGBoost | 3 | 2791 | 0.800 | 0.780 | 0.820 | 1 | 319 | 0.81 | 0.75 | 0.88 |
|  | NNET | 4 | 5095 | 0.740 | 0.730 | 0.760 | 1 | 350 | 0.89 | 0.86 | 0.92 |
|  | RF | 4 | 2946 | 0.720 | 0.700 | 0.740 | - | - | - | - | - |
|  | other | 1 | 141 | 0.900 | 0.810 | 1.00 | - | - | - | - | - |
|  | Overall | 45 | 47311 | 0.760 | 0.740 | 0.78 | 10 | 14975 | 0.79 | 0.75 | 0.83 |

other=κNN、NB、COX

NB：Naïve Bayes；LR：Logistic Regression；DT：Decision Tree；RF：Random Forest；COX：Cox Regression；XGBoost：Extreme Gradient Boosting；

SVM：Support Vector Machine, classifier；NNET：Neural Net；GBM：Gradient Boosted Machine；κNN：κ-Neural Net

**Supplementary File 3：****Data analysis details**
