# Supplementary material for: Machine learning in predicting cardiac surgery-associated acute kidney injury: A systemic review and meta-analysis
Source: Front Cardiovasc Med. 2022 Sep 15;9:951881. doi: 10.3389/fcvm.2022.951881 (PMC9520338; doi:10.3389/fcvm.2022.951881)
Supplement: Supplementary file 4 [file Table_4.DOCX]

**Identification of studies via databases and registers**

Records identified from: Cochrane Library，PubMed，Embase，Web of science：

Databases (n =1909)

Records removed *before screening*:

Duplicate records removed (n =220)

**Identification**

Number of studies excluded(n=292)：

Review(n=69)

Meta-analysis(n=22)

Conference abstract(n=137)

Animal experiments(n=16)

Non-English literature(n=48)

Records screened (n =1689)

Reports sought for retrieval

(n =1397)

Records excluded on the basis of title and abstract criteria （n=1357）

**Screening**

Reports excluded:7

1.Unable to get full text(n=5)

2.Unable to extract data(n=2)

Reports assessed for eligibility

(n =40 )

Article retrieval again and meet the requirements (n=5)

Full-text articles screened for eligibility(n=33)

**Included**

Studies included in qualitative synthesis(n=38)
